# Malaria transmission risk in the city of Accra, Ghana: vector behavior and distribution

**DOI:** 10.21203/rs.3.rs-6008722/v1

**Published:** 2025-02-14

**Authors:** Abdul Rahim Mohammed Sabtiu, Isaac Kwame Sraku, Christopher Owusu-Asenso Mfum, Yaw Akuamoah-Boateng, Richard Tettey Doe, Emmanuel Nana Boadu, Judith Dzifa Azumah, Nana Aba Eyeson, Anisa Abdulai, Godfred Amoateng, Simon Kwaku Attah, Fred Aboagye-Antwi, Yaw Asare Afrane

**Affiliations:** University of Ghana; University of Ghana; University of Ghana; University of Ghana; University of Ghana; University of Ghana; University of Ghana; University of Ghana; University of Ghana; University of Ghana; University of Ghana; University of Ghana; University of Ghana

**Keywords:** Anopheles, urban malaria, sporozoite rate, bloodmeal, Entomological inoculation rate

## Abstract

**Background:**

Urban malaria transmission in sub-Saharan Africa is often underestimated, emphasizing the need for research on vector distribution, abundance, behavior, and infectivity in cities. Since 2003, urban malaria transmission has intensified. Monitoring *Anopheles*populations is crucial for developing effective interventions. This study examined the biting and resting habits, distribution, abundance, and *Plasmodium*infection of malaria vectors in Accra.

**Methods:**

Adult malaria vectors were collected using human landing catch (HLC) and Prokopack aspiration (PPK) at ten sites in Accra, categorized into five groups: sites with Irrigated Urban Farming (IUF), Lower Socioeconomic Status (LS), Middle Socioeconomic Status (MS), High Socioeconomic Status (HS), and Peri-urban (PU) areas. Biting patterns and entomological transmission indices of *An. gambiae* s.l. were assessed, Molecular methods were used to detect sporozoite infection, bloodmeal sources, vector species, and insecticide resistant mutation genes.

**Results:**

A total of 41,747 mosquitoes were collected. Of these, 95.49% (39,863/41,747) were host-seeking collected through human landing catch (HLC), consisting of *Anopheles* (52.54%, 20,945/39,863), *Culex*(42.00%, 16,742/39,863), *Aedes* (3.74%, 1,491/39,863), and Mansonia (1.72%, 685/39,863). The remaining 4.51% (1,884/41,747) were resting mosquitoes collected using a Procopack aspirator, comprising *Anopheles* (31.26%, 589), *Culex* (57.21%, 1,078), and *Aedes* (11.52%, 217). Overall, the irrigated urban farming (IUF) site category recorded the highest abundance of *An. gambiae* s.l. (48.63%, 10,466/21,520), followed by the peri-urban (PU) site category (18.06%, 3,887/21,520), high socioeconomic (HS) site category (16.42%, 3,533/21,520), middle socioeconomic (MS) site category (13.74%, 2,956/21,520), and low socioeconomic (LS) site category (3.15%, 678/21,520) in both HLC and Procopack collections.

Significantly higher mosquito biting activity was observed during the late evening (LE), the classical mosquito biting time, in both the rainy (71.45%, 11,337/15,865) and dry (72.21%, 3,658/5,066) seasons. Indoor An. gambiae s.l. had higher sporozoite infectivity (70.64%, 77/109) than outdoor mosquitoes (29.35%, 32/109) (χ^2^ = 6.78, P = 0.009). Tuba from the IUF site had the highest sporozoite infectivity (32.11%, 35/109), with higher indoor (24.77%, 27/109) than outdoor (7.34%, 8/109) rates.

**Conclusion:**

*Anopheles gambiae* s.l. drives urban malaria transmission in Accra, with high human bloodmeal indices and sporozoite infectivity, especially indoors. Urban agriculture promotes vector proliferation. Targeted indoor control and management of breeding sites in IUF areas are essential to reduce transmission.

## Background

Urbanization is significantly altering malaria vector dynamics worldwide (1), consequently increasing urban malaria transmission in many sub-Saharan African countries (2). Despite this, urban malaria transmission remains underestimated, highlighting the need for research into vector distribution, abundance, and behaviour in urban environments. Rapid and often unplanned urbanization in many cities across sub-Saharan Africa, characterized by the encroachment into lowland areas for housing, inadequate drainage systems, and poor road infrastructure, creates ideal breeding grounds for malaria vectors. Additionally, practices such as irrigated urban farming and the adaptation of *Anopheles* mosquitoes to polluted breeding sites are contributing to increased malaria vector populations in these cities (3)(1).

The fight against malaria in sub-Saharan Africa has benefited significantly from two key interventions: Indoor Residual Spraying (IRS) and Long-Lasting Insecticidal Nets (LLINs) (4). However, in Ghana’s urban areas, these tools face important limitations. IRS programs are confined to rural regions (5), while urban residents are reluctant in LLINs installation and use (6)(7). The urban environment presents additional challenges: widespread use of agricultural chemicals on urban farms, combined with household insecticide sprays and mosquito coils, has led to increase in insecticide resistance in urban malaria vectors (8)(9)(10)(11). Furthermore, urban housing features like screened windows, doors, and eaves have inadvertently pushed mosquitoes to adapt their feeding behavior to outdoors.

In Accra and other African cities, most people reside in urban slums characterized by lower socioeconomic conditions and poor housing infrastructure (12). Due to overcrowding and inadequate ventilation, residents in these areas often spend part of their sleeping hours outdoors. Generally, people in urban areas go to bed later and thus spend more time outside at night compared to those in rural areas. Therefore, this shift is particularly concerning given the common urban practices of outdoor sleeping and nighttime activities, which increase residents’ vulnerability to malaria transmission (13).

Furthermore, given the behavioural plasticity of *An. gambiae* s.l. (14), adult malaria vectors in urban areas are likely to exhibit different behaviours compared to their rural counterparts due to varying ecological and social conditions. Traditional understanding of *Anopheles* mosquito behaviour, including resting and biting patterns, is increasingly complex in urban settings, necessitating a more nuanced investigation. The unique behaviours of mosquitoes in urban landscapes challenge conventional malaria control strategies, underscoring the need to re-evaluate and adapt interventions to address the diverse resting sites favoured by urban vectors (15). Continuous surveillance of urban anopheline populations is essential for gathering critical data to design effective and sustainable interventions aimed at disrupting vector proliferation and malaria transmission in urban areas (2).

This shift in mosquito behavior, along with their adaptation to polluted breeding sites and the emergence of new, resilient vector species that thrive in urban environments, can hinder malaria elimination efforts. Understanding the host preferences, insecticide resistance markers, and the biting and resting behaviors of malaria vectors is crucial for comprehending malaria transmission dynamics and evaluating the effectiveness of control and resistance management strategies(16). Therefore, this study aimed to investigate the biting and resting behavior, as well as the distribution and abundance of adult malaria vectors in Accra.

## Methodology

### Study sites

The study was conducted at ten (10) sites within the city of Accra, the capital of Ghana, and its peri-urban areas. Two (2) sites were randomly selected from areas categorized as low socioeconomic (LS), middle socioeconomic (MS), high socioeconomic (HS) (17)(18), and irrigated urban farming (IUF). Additionally, two (2) sites were selected from the peri-urban areas surrounding Accra.

Sites selected in the irrigated urban farming category were Opeibea (5°35’52.8”N 0°10’48.2”W) and Tuba (5°30’47”N 0°23’16”W). These sites have irrigated vegetable farms in low-lying areas of the city that create all-year-round breeding habitats for the malaria mosquitoes. Furthermore, farmers in these areas use various agricultural chemicals that may contribute to insecticide resistance in mosquitoes. The low socioeconomic (LS) sites were Nima (5°35′0″N 0°12′0″W) and Chorkor (5°31′39″N 0°13′55″W), which are slums characterized by dilapidated and overcrowded structures on poorly demarcated plots, lacking proper drainage and sanitation systems.

The middle socioeconomic (MS) sites were Dansoman (5°33′0″N 0°16′0″W) and Teshie (5°35′0″N 0°6′0″W). These areas have standard housing on well-demarcated plots, with most roads either tarred or untarred, and they possess designed but poorly managed drainage and sanitation systems. High socioeconomic (HS) sites were East Legon (5°38’16.39”N 0°9’40.33”W) and Tantra Hill (5°34’44.0148”N 0°13’46.812”W), feature the highest quality housing in Ghana, with well-planned environments, best-managed drainage, and sanitation systems, and well-maintained roads with few potholes to serve as mosquito breeding habitats.

The peri-urban areas were Oyarifa (5°46′14″N 0°10′50″W) and Medie (5°45’43”N 0°19’20”W). These areas, just outside the city, were characterized by new settlements with many roads and buildings under construction and higher vegetation cover. The land use type in these areas creates numerous breeding habitats during the rainy season. Differences in landscape, drainage systems, sanitation, and land use (agriculture/non-agriculture) among these sites will aid in understanding their impact on urban malaria transmission and mosquito resistance to insecticides.

Accra, within the Greater Accra region, had a total population of about 2,557,000 in 2021, a population growth rate of 24.13% from 2010, and a land area of 225.67 km^2^ (19). The region experiences two rainy seasons, with an average annual rainfall of 730 mm and an average temperature of 27.6°C. The relative humidity is generally high, ranging from 65% in the midafternoon to 95% at night. The rainfall pattern, combined with poor drainage, supports the formation of stagnant waters, while the temperature and humidity create a favorable environment for mosquitoes.

### Collection of host-seeking mosquitoes.

The human landing catch (HLC) method was employed to collect host-seeking mosquitoes. Twelve (12) houses were selected from each study site for this collection during each season (dry and rainy). Each day, outdoor of four (4) houses were chosen for three consecutive nights. Two trained volunteers at each house were stationed outdoors in the dark from 6:00 PM to 6:00 AM to catch mosquitoes landing on their legs. Each collector was equipped with a falcon tube and a flashlight to assist with mosquito capture. To attract mosquitoes, collectors exposed their lower limbs to increase the skin surface area. Using the flashlight, the collectors trapped mosquitoes that landed on their skin by guiding them into the falcon tubes. Once a mosquito entered the tube, the collector quickly covered the open end with their thumb and transferred the mosquito into a pre-labeled collection cup. Different locations were chosen each night to ensure a representative *Anopheles* vector population for the study site (20).

To ensure the quality of collections, supervisors conducted regular rotations between different groups throughout the night. Mosquitoes captured each hour were either killed by placing them in a −20°C freezer or exposing them to chloroform. They were then stored in individual tubes with silica gel, labeled with study site, date and time of collection, and transported to the laboratory for identification. Mosquito biting patterns were classified into the following categories: early evening (EE) (18:00–21:00), late evening (LE) (00:00–04:00), and early morning (EM) (05:00–06:00).

### Collection of indoor and outdoor resting mosquitoes using Prokopack aspirators

Resting mosquitoes, both indoors and outdoors, were collected using Prokopack (PPK) aspirators from 15 houses per day at each site. Sampling was conducted each morning from 5:30 AM to 8:00 AM over a three-day period. Households were requested the night before the sampling not to open their doors and windows when they wake up the next morning to avoid mosquitoes exiting. Adult mosquitoes were then sampled each morning of collection (5:30am to 8:00am) using the prokopack aspirator. The aspirator was used to hoover the ceiling, under tables and beds, wall and all possible resting surfaces. Aspirated mosquitoes were then put into holding cups and transported to the laboratory for identification and further processing (21).

### Mosquito Species Identification

All captured mosquitoes were sorted into genera (*Anopheles*, *Culex* and *Aedes*), sex and gonotrophic stages (unfed, fully fed, semi-gravid, and gravid), and *An. gambiae* s.l. mosquitoes were identified based on morphological characteristics under a stereomicroscope (Olympus, SZ60, Japan) using the identification keys developed by Maureen Coetzee (22). Identification of sibling species within the *Anopheles gambiae s.l.* complex was achieved through rDNA polymerase chain reaction (PCR) (23). Furthermore, the specific Identification of *An. gambiae* s.s. and *An. coluzzii* was carried out using PCR-based restriction fragment length polymorphism (RFLP) analysis (24).

### Genotyping for kdr and ace-1 mutations

To genotype for the kdr mutation, DNA was extracted from mosquito legs using the ZR DNA MicroPrep kit (Zymo Research), following the manufacturer’s instructions. Standard PCR assays were employed to detect the presence of the L1014F and L1014S kdr alleles, using a modified protocol as described by (25). Additionally, the G119S mutation in the ace1 gene was assessed through PCR analysis, following the protocol outlined by (26).

### Blood meal analysis in resting Anopheles gambiae mosquitoes

The abdomens of the blood-fed resting *Anopheles* mosquitoes were cut into transverse sections. Genomic DNA was extracted from the mosquito abdomens using the ZR DNA MicroPrep kit (Zymo Research, CA) following the manufacturer’s instructions. One universal reverse primer and five animal-specific (human, cow, goat, pig, and dog) forward primers were used for amplification of the mitochondrial cytochrome b gene to test for specific host blood meal origin using conventional PCR (27). Positive controls were included for each host in the PCR analyses, and laboratory-reared unfed An. gambiae were used as the negative control.

### Detection of sporozoites

DNA extracted from the head and thorax of each mosquito sample was analyzed for the presence of *Plasmodium falciparum* sporozoites using polymerase chain reaction (PCR) following the method described by Echeverry et al. (28). This assay was conducted on all resting *Anopheles gambiae s.l.* (n = 589) collected, as well as a subsample of host-seeking *An. gambiae s.l.* (n = 2,472) representing samples from all study sites in both seasons.

### Data Analysis

Descriptive analysis was performed to compare adult mosquito abundance between different study sites, and indoor and outdoor. This was presented in the form of tables, bar charts and graphs. The association of Blood meal sources, sporozoites infection rate and the abundance of malaria vectors resting indoor and outdoor as well as across sites were tested using Chi-square. The sporozoite infection rate (IR) was expressed as the proportion of mosquitoes positive for Plasmodium sporozoite, that is the number of sporozoite positive mosquitoes divided by total number of mosquitoes tested. Human Biting Rate (HBR) was calculated as the total number of mosquitoes collected by HLC per the total number of collectors per nights collected. The Entomological Inoculation Rates (EIR) was calculated as the product of the human biting rate and the sporozoite rate. Analysis of Variance (ANOVA) test was performed to compare the means of the biting categories across seasons and sites. The kdr-w (L1014F), kdr-e (L1014S) and Ace-1 (G119S) mutation frequencies were calculated according to the following formula: F (kdr) = (2RR + RS) / 2n, where RR is the number of homozygote resistant mosquitoes, RS is the number of heterozygotes resistant mosquitoes, and n is the total number of mosquitoes analysed (29). The human blood index (HBI) and bovine blood index (BBI) was calculated as the proportion of Anopheles mosquitoes that had taken a blood meal from human or other vertebrates respectively. Maps were downloaded from google and modified with Adobe Photoshop CS6 to show locations of study sites

### Ethical clearance

This study was granted scientific and ethical approval by the Ethical and Protocol Review Committee (EPRC) of the College of Health Sciences, University of Ghana, Korle-Bu Campus. Informed consent (verbal and written) was acquired from opinion leaders of selected study sites and house/household heads. To reduce the risk of mosquito bites collectors were trained to catch mosquitoes before piercing their proboscis. To get the support of residence and safety supervision within the night volunteers were selected from study sites.

## Results

### Seasonal distribution and abundance of host-seeking malaria vectors.

A total of 39,863 mosquitoes consisting of four different genera were collected using HLC across all study sites during the sampling period: *Anopheles* (52.54%, 20,945/39,863), *Culex* (42.10%, 16,742/39,863), *Aedes* (3.74%, 1,491/39,863) and *Mansonia* (1.72%, 685/39,863). The dry season recorded 41.75% (16,642/39,863) of mosquitoes collected [*Anopheles* (30.47%, 5,071/16,642), *Culex* (64.46%, 10,727/16,642), *Aedes* (1.44%, 240/16,642), *Mansonia* (2.55%, 424/16,642)] and 58.25% (23,221/39,863) in the rainy season [*Anopheles* (68.36%, 15,874/23,221), *Culex* (25.90%, 6,015/23,221), *Aedes* (4.61%, 1,071/23,221), *Mansonia* (1.12%, 261/23,221)]. Overall, *Anopheles* dominated (52.54%, 20,945/39,863), followed by *Culex* (42.00%, 16,742/39,863), *Aedes* (3.74%, 1,491/39,863), and *Mansonia* (1.72%, 685/39,863). Among the *Anopheles*, the highest abundance was *An. gambiae* s.l. (99.93%, 20,931/20,945), with smaller proportions of *An. funestus* s.l. (0.02%, 4/20,945), *An. pharoensis* (0.03%, 6/20,945), and *An. ru pes* (0.02%, 4/20,945) (Table 1).

Among the site categories, irrigated urban farming (IUF) had the highest abundance of *An. gambiae* s.l. (48.85%, 10224/20931), followed by peri-urban (PU) (17.86%, 3738/20931), and the lowest was from low socioeconomic (LS) (2.84%, 595/20931). The rainy season had a higher abundance of *Anopheles gambiae* s.l. in all sites categories as compared to the dry season [IUF (dry = 3,067, rainy = 7,157), PU (dry = 805, rainy = 2,933), LS (dry = 127, rainy = 468), MS (dry = 469, rainy = 2,402) and HS (dry = 598, rainy = 2,905)]. Among the individual sites, Tuba in the irrigated urban farming category had by far the highest abundance of *An. gambiae* s.l. in both dry and rainy season [dry (47.55%, 2,409/ 5,066), rainy (38.20%, 6,061/15,865)], whereas Chorkor in the LS categories had the lowest abundance of mosquitoes in both seasons [dry (1.11%, 56/ 5,066), rainy (1.32%, 210/15,865)]. *An. funestus* species were exclusively collected from Medie in the peri-urban site category (Table 1) during the dry season.

Among the sampled Anophelines collected, only 0.07% (14/20,945) comprised of other *Anopheles* species (Table 1). These species consist of *An. rufipes* (28.57%, 4/14), *An. funestus* (28.57, 4/14) and *An. pharoensis* (42.86%, 6/14). *An. pharoensis* species were exclusively collected during the rainy season, whereas, *An. funestus* on the other hand were sampled during the dry seasons.

### Seasonal distribution and abundance of resting malaria vectors

A total of 1,884 resting mosquitoes were collected from all sites. The most abundant genera of mosquitoes sampled were the culicines (57.22%, 1,078/1,884), followed by the Anophelines (31.26%, 589/1,884) and then Aedines (11.52%, 217/1,884). More *Anopheles gambiae* s.l. were sampled during the rainy season (77.08%, 454/589) as compared to the dry season (22.92%, 135/589) (t = −0.0405, df = 587, *P* = 0.48). Tuba from the irrigated urban farming site category had the highest abundance of resting *An. gambiae* s.l., 26.49%, 156/589 (rainy = 116, dry = 40), with the least sampled from East Legon from the HS site category, 1.70%, 10/589 (rainy = 7, dry = 3).

### Indoor and outdoor abundance of resting Anopheles gambiae s.l.

Of the total (n = 589) resting *Anopheles gambiae* s.l. mosquitoes that were sampled during the study period, significantly abundant *Anopheles gambiae* s.l were collected indoors (60.44%, 356/589) as compared to outdoors (49.56%, 233/589) (t = 1.9103, df = 587, *P* = 0.03). Among the sites, Tuba (IUF) had the highest abundance of *An. gambiae* s.l. in both indoor (24.72%, 88/356) and outdoor (29.18%, 68/233), whereas the least indoor (1.40%, 5/356) and outdoor (2.15%, 5/233) abundance were recorded in East Legon (HS) (Table 2).

### Species discrimination in host-seeking Anopheles gambiae complex

From the host-seeking *An. gambiae* s.l. collected, a subsample of 2,232 from all the study sites in both seasons were randomly selected and used to discriminate the sibling species. *An. gambiae* s.s (68.86%, 1,537/2,232) was the most abundant species followed by *An. coluzzii* (27.24%, 608/2,232), and hybrids of *An. gambiae s.s*. and *An. coluzzii* (3.90%, 90/2,232) (Table 3). With respect to socioeconomic characterization, more *An. coluzzii* was collected from LS [*An. gambiae* s.s. = 26.14% (40/153); *An. coluzzii* = 67.97% (104/153); hybrids = 5.88% (9/153)] and MS [ *An. gambiae* s.s.= 36.65% (103/281); *An. coluzzii* = 62.28% (175/281); hybrids = 1.07% (3/281)] site categories. However, *An. gambiae* s.s. was more abundant in the other site categories [PU, HS, IUF] (Tables 3).

### Species discrimination in resting Anopheles gambiae complex

**Figure 2:**
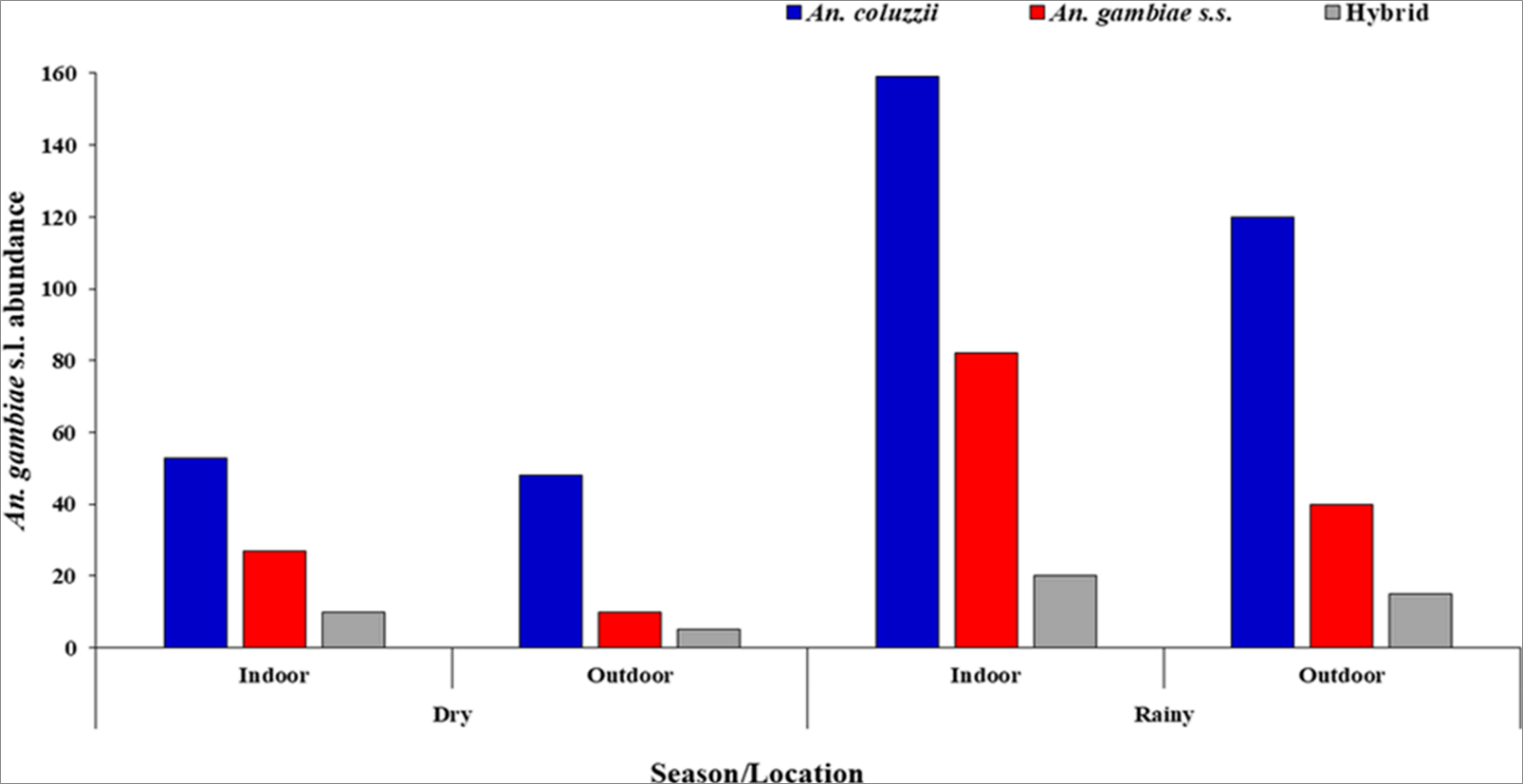
Indoor and outdoor seasonal distribution of resting *Anopheles gambiae* species

With resting mosquitoes, the total *An. gambiae* s.l. (n = 589) from all the study sites were used to discriminate the sibling species. *An. coluzzii* (64.52%, 380/589) were the most abundant, followed by *Anopheles gambiae* s.s, (26.99%, 159/589) and then Hybrids of *An. coluzzii* and *An. gambiae* s.s. (8.49%, 50/589). During both seasons, *An. coluzzii* were the most abundant sampled, Dry [indoor (48); outdoor (41)]; Rainy [indoor (164); outdoor (127)], followed by *An. gambiae* s.s, Dry [indoor (24); outdoor (9)]; Rainy = [indoor (85); outdoor (41)], and then hybrids; Dry [indoor (8); outdoor (5)] Rainy = [indoor (22); outdoor (15)], (Fig. 2).

### Biting times of An. gambiae s.l. in the study sites

**Figure 3:**
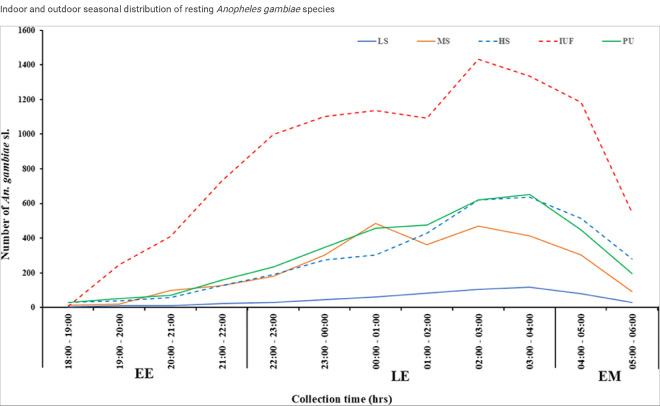
Biting patterns of *An. gambiae* s.l according to site categories

The biting patterns of *Anopheles* mosquitoes were categorized as early evening (EE) from 18:00 to 22:00 hours, late evening (LE) from 22:00 to 04:00 hours, and early morning (EM) from 04:00 to 06:00 hours. *An. gambiae* s.l. exhibited the highest biting activity during the late evening (LE) at 71.64% (14,995/20,931), followed by the early morning (EM) at 17.56% (3,675/20,931), and the lowest during the early evening (EE) at 10.80% (2,261/20,931). This pattern was consistent across all sites and site categories. Notably, significantly higher biting activity was recorded in the LE for both the dry (72.21%, 3,658/5,066) and rainy seasons (71.45%, 11,337/15,865) [F (2, 27) = 6.03, P = 0.019, 95% CI 135.2691–1388.731] (Fig. 3).

Biting activity varied across site categories, with a non-significant trend of higher activity observed in the irrigated urban farming site category, particularly during the late evening, peaking between 02:00 and 03:00 hours [F (4, 10) = 1.36, P = 0.176, 95% CI −793.9493–3781.949].

The human-biting activity of *An. gambiae* s.l. displayed a similar pattern across all study sites, showing a bimodal distribution. The first and highest peak occurred between 02:00 and 03:00 hours (15.52%, 3,248/20,931), while a secondary peak was observed in the early morning between 04:00 and 05:00 hours (12.09%, 2,530/20,931). Although biting activity higher in the early morning hours compared to the early morning hours, biting activity steadily declined from 05:00 to 06:00 hours.

### Blood meal sources of resting An. gambiae s.l. mosquitoes collected

Out of the 589 *An. gambiae s.l.* mosquitoes collected, 70.63% (416/589) were found to have fed on blood from various sources. The Human Blood Index (HBI) was consistently higher across all site categories and species at 86.30% (359/416), compared to the Bovine Blood Index (BBI), which was 29.33% (122/416). Hybrids collected indoors at the Peri-Urban (PU) site exhibited the highest HBI of 100% (2/2), while the highest BBI, 50% (5/10), was observed among hybrids collected outdoors at the Irrigated Urban Farming (IUF) site.

Mixed blood meal sources were recorded sporadically, with the highest occurrences at irrigated urban farming and peri-urban site categories. *An. Gambiae s.s.* exhibited consistently high HBI across all site categories, particularly for indoor feeding: LS (indoor = 57%, outdoor = 57%), MS (indoor = 52%, outdoor = 100%), HS (indoor = 100%, outdoor = 0%), IUF (indoor = 54%, outdoor = 46%), and PU (indoor = 82%, outdoor = 79%).

Similarly, *An. coluzzii* demonstrated strong anthropophilic tendencies with high HBI across all site categories: low socioeconomic (indoor = 66%, outdoor = 65%), middle socioeconomic (indoor = 47%, outdoor = 0%), high socioeconomic (indoor = 100%, outdoor = 72%), irrigated urban farming (indoor = 52%, outdoor = 45%), and peri-urban (indoor = 88%, outdoor = 68%) (Table 4).

### Sporozoite infection rates in Anopheles gambiae s.l.

All the resting *Anopheles gambiae* s.l sampled (n = 589) were analyzed for the presence of *Plasmodium falciparum* circumsporozoite protein (CSP). Of the 18.51% (109/589) that tested positive, those collected from indoors had higher sporozoite infectivity (21.94%, 77/351) as compared to those collected from outdoors (13.45%, 32/238) (*X*^2^ = 6.7818, df = 1, *P* = 0.009). Tuba from the IUF site category recorded the highest frequency of CSP positive *Anopheles gambiae* s.l. (32.11%, 35/109), [indoor = 24.77% (27/109); outdoor = 7.34% (8/109)]. And the least was recorded in East Legon (1.83%, 2/109) [indoor = 1.83% (2/109); outdoor = 0% (0/109)] (Table 4).

A subsample of 2,472 [dry = 1,670; rainy = 802] *An. gambiae* s.l. mosquitoes collected through HLC were also tested for *Plasmodium falciparum* circumsporozoite (CSP). Overall, fty-four (54) [dry = 21/1,670; rainy = 33/802] (χ^2^ = 14.8, df = 13, *P* = 0.320) were positive for *P. falciparum* CSP representing a 0.013 and 0.041 infection rate respectively. Overall, the highest sporozoite rate was recorded in Dansoman (MS) (0.058) and Medie (PU) (0.043), whereas the least was recorded in Tantra hill (HS) (0.005). Among the sites categories, sporozoite rates did not vary significantly (χ^2^ = 59.0, df = 52, *P* = 0.235) (Table 5).

### Entomological inoculation rate of host-seeking An. gambiae s.l.

The average EIR recorded in this study was 1.526 infective bites per person per night (ib/m/n). The EIR for the dry and rainy seasons was 0.191 (ib/m/n) and 2.862 (ib/m/n) (χ^2^ = 18.80, df = 15, *P* = 0.331) respectively. Overall, during the rainy season, it is expected that an unprotected individual in Tuba is estimated to receive an averagely high infective bite of 0.409 (ib/m/n) with the least in Tantra Hill, Dansoman, Teshie and Nima (0 ib/m/n). However, in the dry season, it is expected that an unprotected individual in Oyarifa is expected to receive a high infective bite of 0.602, 0.5 and 0.41 respectively. Furthermore, a monthly estimated EIR (estEIR) of 45.719 ib/m/n was calculated for all the study sites, which translated to 695.37 infective bites/man/year. (Table 6).

### Insecticide resistance mutation genotypes in resting Anopheles gambiae s.l.

All *Anopheles gambiae s.l.* samples (n = 589) collected from indoor and outdoor environments were genotyped to detect insecticide resistance mutations: L1014F, L1014S, and G119S *Ace-1*.

The L1014F allele was present at a frequency of 100% across indoor and outdoor environments and all *An. gambiae s.l.* species, except in *An. gambiae s.s.*, where it was observed at a slightly lower frequency of 90%. The L1014S mutation was detected at a very low frequency (10%) and was limited to *An. gambiae s.s.* and hybrids.

For the G119S *Ace-1* mutation, the allele frequency was 70% in indoor samples and 60% in outdoor samples. Among the species, G119S was observed at a frequency of 70% in both *An. coluzzii* and *An. gambiae s.s.*, and 50% in hybrids (Table 7).

## Discussion

To effectively control and eliminate malaria in urban environments, it is essential to thoroughly understand vector-human interactions and resting behaviors in these areas (15). The intricate relationship between the spatiotemporal distribution and biting patterns of malaria vectors is crucial for designing targeted control measures. This study reveals notable patterns in the resting and host preference behaviors of urban malaria vectors, while also examining biting patterns and the infectivity rate of *Plasmodium falciparum* sporozoites in Accra, Ghana. The findings indicate that malaria vectors show higher resting densities indoors, with a significantly higher Human Blood Index (HBI) observed in indoor *Anopheles* mosquitoes compared to those resting outdoors. These insights are vital for developing effective malaria control strategies in urban settings.

*Anopheles gambiae* s.l. was found to be more abundant during the rainy season compared to the dry season in all collections. This is obviously due to the increased availability of breeding habitats during this season that facilitate oviposition by gravid females. This observation aligns with several studies in Ghana indicating that malaria vectors are more abundant in the rainy season (3)(29). While the rainy season generally provides more breeding opportunities for *Anopheles*, the dry season can support higher mosquito populations in certain areas, particularly those with human intervention and stable water sources, such as waterlogged and irrigated agricultural sites (53).

In this investigation, a significant higher abundance of *Anopheles* mosquitoes resting indoors compared to outdoors was recorded. Typically, mosquitoes prefer dark and humid environments for resting, such as chicken coops, open pots, and animal sheds. The lower number of *Anopheles* mosquitoes found outdoors may be attributed to the scarcity of such structures in urban areas. A similar finding was reported by Govella *et al*. in urban Dar es Salaam, where the mean catch of indoor resting *An. gambiae s.l.* was higher than that outdoors (30). In contrast, studies from Chennai, India, reported a greater abundance of outdoor resting malaria vectors (31). This suggests that the resting behavior of malaria vectors, whether indoors or outdoors, may be influenced by the availability of suitable resting structures.

While *An. coluzzii* was the most abundant vector among the indoor and outdoor resting *An. gambiae* s.l., *An. gambiae s.s.* was the most frequently recorded vector among host-seeking *An. gambiae* s.l.. Notably, *An. coluzzii* dominated in the low socioeconomic (LS) sites (Nima and Chorkor) and middle socioeconomic (MS) sites (Teshie and Dansoman). These locations are characterized by a diverse array of habitats heavily polluted with organic matter. The prevalence of *An. coluzzii* in these areas may be related to the stability of the breeding habitats, suggesting a preference for permanent larval sites. Its abundance and distribution remain consistent regardless of seasonal changes or rainfall patterns. Similar results have been documented in Ghana (32)(33) and Benin (34). Additionally, this study noted the presence of secondary malaria vectors, which have been linked to malaria transmission in other regions (35) (36)(37).

Blood meal analysis in this study indicated that malaria vectors predominantly fed on animal blood rather than human blood. The Human Blood Index (HBI) was higher in indoor resting Anopheles mosquitoes, while the Bovine Blood Index (BBI) was elevated in outdoor resting mosquitoes. Urban homes, typically well-screened to block mosquito entry, may lead some vectors to seek animal blood sources outdoors. This trend aligns with findings from Akuoko et al, (38) in Ghana, where a higher HBI was observed indoors despite interventions like IRS and LLIN, as well as Machani et al.(39) in Kenya. In contrast, Bedasso et al. (40) reported a higher HBI in outdoor resting mosquitoes in Ethiopia. The mixed blood meal sources observed among the same *Anopheles* species indicate flexible feeding behavior in *An. gambiae* s.l., consistent with ndings by Orsborne et al.(14).

This study found that outdoor *An. gambiae* s.l. mosquitoes prefer to bite late at night, coinciding with when people are asleep. Outdoor sleeping is a significant factor contributing to this late-night biting peak, as individuals in the urban settings often stay outside during the early evening due to high indoor temperatures and return inside around 02:00 hours when it cools down. Activities such as funerals, church gatherings, and trading also keep people outdoors late (41). This finding corroborates with a study in Ghana (20) and Uganda (42) which reported peak biting times between 23:00 and 05:00 hours. Additionally, early morning biting activity may occur as household members begin their chores around 03:00 hours, exposing them to malaria risk when they are unprotected by LLINs and IRS (43).

Sporozoite rates determined during the study did not vary significantly among seasons. This finding may suggest that malaria transmission did not change between the seasons. The infection rates found in outdoor biting mosquitoes could suggest ongoing residual malaria transmission regardless of the vector control tools employed in the study sites. However, among the resting mosquitoes sampled, a higher sporozoite infection rate was observed in indoor mosquitoes compared to outdoor resting mosquitoes across all study sites. This could have resulted from the higher HBI observed among indoor resting mosquitoes in this study.

The entomological inoculation rate (EIR) is the preferred tool for assessing malaria endemicity and transmission intensity (44)(45). Studies across Africa have shown that EIR estimates in rural areas range from 0 to 884 infectious bites per person annually, while urban environments typically report lower EIRs, ranging from 0 to 43 infectious bites per person annually(46)(47). This study recorded a significantly higher urban annual EIR of *An. gambiae s.l.* compared to the average urban EIRs reported across sub-Saharan Africa. The results of this study indicated that the risk of malaria transmission is high in Tuba, an irrigated urban farming (IUF) site, and that residents who have little to no protection against malaria vectors may be exposed to up to 992.64 infectious bites per year. The annual estimated EIR of *An. gambiae* s.l. reported in this study (557.00 ib/y) was significantly higher than the EIRs reported in three cities in Senegal—Diourbel (3.65), Touba (7.31), and Kaolack (40.21)(48), as well as in Dodowa (21.9) within Ghana’s coastal forest zone(49). Interestingly, similarly high annual EIRs (584.2 and 607.5) were reported in certain urban areas of Cameroon (50).

The presence of insecticide resistance mutations in *Anopheles gambiae s.l.* populations highlight the ongoing challenge of controlling malaria vectors in urban and rural settings. This study provides critical insights into the frequency and distribution of three key resistance mutations—L1014F, L1014S, and G119S Ace-1—among *An. gambiae s.l.* collected from both indoor and outdoor resting sites.

The L1014F mutation, associated with resistance to pyrethroids and DDT, was observed at a near-universal frequency (100%) in most *An. gambiae s.l.* populations sampled indoors and outdoors. This consistent presence across locations and species underscores the widespread xation of this mutation in the population, likely driven by extensive insecticide pressure from indoor residual spraying (IRS), long-lasting insecticidal nets (LLINs), and also the use of insecticide aerosol spray and some agrochemicals. However, a slightly lower frequency (90%) was detected in *An. gambiae s.s.*, suggesting possible differences in selective pressure or genetic variability within this species. The dominance of L1014F in *An. gambiae s.l.* populations align with findings from other studies in sub-Saharan Africa (9) (20) (38) (51), where pyrethroid resistance is becoming increasingly prevalent.

In contrast, the L1014S mutation which is prevalent in East Africa, was detected at a very low frequency (10%) and was limited to *An. gambiae s.s.* and hybrids. This mutation, which also confers resistance to pyrethroids and DDT, appears to be less widespread in the study population. Its low frequency may be attributed to its relatively recent emergence, lower fitness advantages compared to L1014F, or geographic variations in insecticide use. Interestingly, findings from Niger by Soumaila et al.(52) reported comparable frequencies of the L1014F and L1014S mutations, ranging from 46–81% and 41–87%, respectively. This suggests that in some regions, both mutations may coexist at significant frequencies, likely driven by local environmental pressures and insecticide application practices. The marked difference in the prevalence of L1014S between this study and Niger highlights the importance of regional surveillance to understand the distribution and dynamics of insecticide resistance mutations across different ecological and operational contexts.

The G119S Ace-1 mutation, linked to organophosphate and carbamate resistance, exhibited a moderate frequency in the population. The allele was more prevalent in indoor samples (70%) compared to outdoor samples (60%), possibly reflecting greater exposure of indoor populations to organophosphate-based insecticides. Among species, the mutation was observed at equal frequencies (70%) in *An. coluzzii* and *An. gambiae s.s.*, with hybrids showing a lower frequency (50%). This pattern indicates variability in resistance development across species and the potential in fluence of ecological or behavioral factors, such as resting and feeding preferences. A similar pattern has been documented in other studies where G119S coexisted with L1014F and/or L1014S mutations within the same populations (9) (20) (38) (51) (52). This co-occurrence indicates a multifaceted adaptation to multiple insecticide classes, highlighting the genetic plasticity of *An. gambiae s.l.* populations in response to selective pressure.

## Conclusion

This study revealed that malaria vector abundance and biting activity in Accra vary by season and location, with *Anopheles gambiae* s.s. being the most common species, and *An. coluzzii* predominating in areas with polluted, permanent larval habitats. The presence of mixed blood meals in resting mosquitoes suggests a risk for zoonotic disease transmission. The study also highlighted an increased likelihood of indoor malaria transmission, indicated by high Human Blood Index (HBI) and Sporozoite Infection Rate (SIR) in resting *An. gambiae* s.l. Additionally, the reported annual entomological inoculation rate (EIR) was significantly higher than typical urban averages, signaling a high malaria transmission risk in Accra, especially near irrigated urban farming areas. To improve malaria control in urban settings, continuous vector surveillance and monitoring are crucial for understanding vector dynamics and informing targeted interventions.

## Figures and Tables

**Figure 1:**
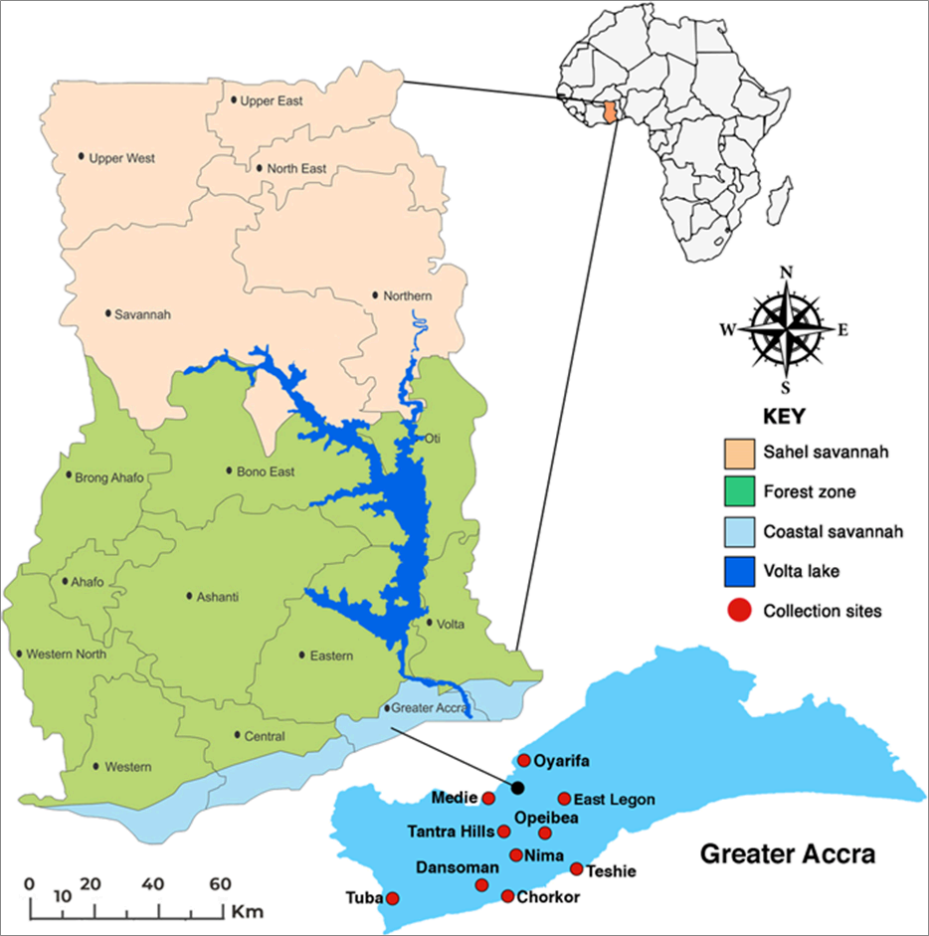
Map of Ghana and Accra showing location of the study sites ((**Source:** https://www.google.com/search?q=Ghana+map&sca, 2023).

**Table 1:** Distribution and abundance of host-seeking mosquito species collected from all sites

	Nima (%)	Chorkor (%)	Teshie (%)	Dansoman (%)	E. Legon (%)	T. Hill (%)	Opeibea (%)	Tuba (%)	Oyarifa (%)	Medie (%)	Total (%)
*Aedes*	25(2.19)	72(6.79)	21(0.55)	20(0.44)	110(3.22)	61(1.31)	37(1.40)	1024(9.55)	84(2.15)	37(0.93)	1491(3.74))
*Culex*	777(67.98)	718(67.74)	1965(51.89)	3397(75.27)	1461(42.71)	2842(61.03)	825(31.12)	999(9.32)	1293(33.04)	2465(61.66)	16742(42.00)
*Mansonia*	12 (1.05)	4(0.58)	12(0.32)	9(0.20)	76(2.22)	23(0.49)	35(1.32)	226(2.11)	275(7.03)	13(0.33)	685 (1.72)
*An. gambiae s.l.*	329 (28.78)	266(25.09)	1789(47.24)	1082(23.97)	1774(51.86)	1729(37.13)	1754(66.16)	8470(79.02)	2260(57.74)	1478(36.97)	20931(52.51)
*An. funestus*	0	0	0	0	0	0	0	0	0	4(0.10)	4(0.01)
*An. rufipes*	0	0	0	1(0.02)	0	0	0	0	2(0.05)	1(0.03)	4(0.01)
*An. pharoensis*	0	0	0	4(0.09)	0	2(0.04)	0	0	0	0	6(0.02)
Total	1143(100)	1060(100)	3787	4513(100)	3421(100)	4657(100)	2651(100)	10719(100)	3914(100)	3998(100)	39863(100)

LS = Lower socioeconomic, MS = Middle Socioeconomic, HS = High Socioeconomic, IUF = Irrigated Urban Farming, PU = Peri-urban.

**Table 2:** Seasonal, indoor and outdoor distribution and abundance of resting *An. gambiae* s.l. from all sites

	Dry			Rainy		
Site Category	Study sites	Indoor (%)	Outdoor (%)	Indoor (%)	Outdoor (%)	Total (%)
LS	Nima	7 (7.95)	4 (6.45)	25 (9.33)	16 (9.36)	52 (8.83)
	Chorkor	5 (5.68)	4 (6.45)	13 (4.85)	9 (5.26)	31 (5.26)
MS	Teshie	7 (7.95)	5 (8.06)	22 (8.21)	14 (8.19)	48 (8.15)
	Dansoman	6 (6.82)	3 (4.84)	28 (10.45)	0 (0.00)	37 (6.28)
HS	East legon	1 (1.14)	2 (3.23)	4 (1.49)	3 (1.75)	10 (1.70)
	Tantra Hill	2 (2.27)	2 (3.23)	10 (3.73)	6 (3.51)	20 (3.40)
IUF	Opeibea	16 (18.18)	11 (17.74)	37 (13.81)	22 (12.87)	86 (15.11)
	Tuba	26 (29.55)	18 (29.03)	62 (23.13)	50 (29.24)	156 (26.49)
PU	Oyarifa	11 (12.5)	7 (11.29)	32 (11.94)	24 (14.05)	74 (12.56)
	Medie	7 (7.95)	6 (9.68)	35 (13.06)	27 (15.79)	75 (12.73)
	Total	88 (100)	62 (100)	268 (100)	171 (100)	589 (100)

**Table 3:** Species discrimination of host-seeking An. gambiae s.l. from all sites

	LS		MS		HS		IUF		PU		
Species	Nima (%)	Chorkor (%)	Teshie (%)	Dansoman (%)	E. Legon (%)	T. Hill (%)	Tuba (%)	Opeibea (%)	Oyarifa (%)	Medie (%)	Total (%)
*An. gambiae s.s.*	34(40.48)	6 (8.70)	38(26.21)	65(47.79)	243 (87.73)	126(50.81)	169(53.82)	231(87.17)	477(93.90)	148(79.57)	1537(68.77)
*An. coluzzii*	47(55.95)	57(82.61)	107(73.79)	68(50)	21(7.58)	108(43.55)	121(38.54)	30(11.32)	20(3.94)	29(15.59)	608(27.20)
Hybrid	3(3.57)	6(8.70)	0(0)	3(2.21)	13(4.69)	14(5.65)	24(7.64)	4(1.51)	11(2.17)	9(4.84)	90(4.03)
Total	84(100)	69(100)	145(100)	136(100)	277(100)	248(100)	314(100)	265(100)	508(100)	186(100)	2235(100)

LS = Lower socioeconomic, MS = Middle Socioeconomic, HS = High Socioeconomic, IUF = Irrigated Urban Farming, PU = Peri-urban.

**Table 4:** Blood meal source of resting An. gambiae s.l. mosquitoes from site categories

Site Category	Blood-meal origins	*An. gamblae* s.s		*An. coluzzii*		Hybrid	
		Indoor (%)	Outdoor (%)	Indoor (%)	Outdoor (%)	Indoor (%)	Outdoor (%)
LS	Number tested	7	7	35	31	0	1
	Human	4 (57)	4 (57)	18 (51)	14 (45)	0	0
	Bovine	0	0	1 (3)	0	0	0
	Dog	0	0	0	0	0	0
	Human + Bovine	0	0	2 (6)	5 (16)	0	0
	Human + Dog	0	0	3 (9)	1 (3)	0	0
	Unidentified	3 (43)	3 (43)	11 (31)	11 (36)	0	1 (100)
	**HBI**	**57**	**57**	**66**	**65**	**0**	**0**
	BBI	0	0	9	16	0	0
MS	Number tested	25	4	37	12	0	0
	Human	8 (32)	4 (100)	16 (43)	12 (100)	0	0
	Bovine	0	0	0	0	0	0
	Dog	0	0	0	0	0	0
	Human + Bovine	2 (8)	0	4 (11)	0	0	0
	Human + Dog	3 (12)	0	0	0	0	0
	Unidentified	12 (48)	0	17 (46)	0	0	0
	**HBI**	**52**	**100**	**47**	**0**	**0**	**0**
	BBI	8	0	9	0	0	0
HS	Number tested	9	2	5	7	1	1
	Human	5 (56)	0	4 (80)	5 (72)	0	0
	Bovine	0	0	0	1 (14)	0	0
	Dog	0	0	0	1 (14)	0	1 (100)
	Human + Bovine	0	0	1 (20)	0	0	0
	Human + Dog	4 (44)	0	0	0	1 (100)	0
	Unidentified	0	2 (100)	0	0	0	0
	**HBI**	**100**	**0**	**100**	**72**	**100**	**0**
	BBI	0	0	20	14	0	0
IUF	Number tested	28	13	90	77	23	10
	Human	12 (43)	6 (46)	43 (48)	26 (34)	8 (35)	0
	Bovine	1 (3)	4 (31)	13 (15)	13 (17)	5 (22)	5 (50)
	Dog	0	0	1 (1)	6 (8)	0	2 (20)
	Human + Bovine	3 (11)	0	2 (2)	5 (6)	2 (9)	1 (10)
	Human + Dog	0	0	2 (2)	4 (5)	2 (9)	0
	Unidentified	12 (43)	3 (23)	29 (32)	23 (30)	6 (26)	2 (20)
	**HBI**	**54**	**46**	**52**	**45**	**52**	**10**
	BBI	14	31	17	23	18	50
PU	Number tested	43	24	41	34	2	5
	Human	33 (77)	17 (71)	24 (59)	20 (59)	2 (100)	4 (80)
	Bovine	0	0	0	0	0	0
	Dog	0	0	0	0	0	0
	Human + Bovine	2 (5)	2 (8)	7 (17)	2 (6)	0	0
	Human + Dog	0	0	5 (12)	1 (3)	0	0
	Unidentified	8 (19)	5 (21)	5 (12)	11 (32)	0	1 (20)
	**HBI**	**82**	**79**	**88**	**68**	**100**	**80**
	BBI	5	8	17	6	0	0

**Table 5:** Sporozoite infection rates detected in resting *Anopheles gambiae* s.l. in all study sites

	Indoor			Outdoor		
Study sites	No. tested	Pf CSP positive	Sporozoite rate (%)	No. tested	Pf CSP positive	Sporozoite rate (%)
Chorkor	18	5	27.78	13	2	15.38
Dansoman	34	7	20.59	3	0	0.00
E. Legon	5	2	40.00	5	0	0.00
Medie	42	7	16.67	33	3	9.09
Nima	26	2	7.69	26	5	19.23
Opeibea	53	3	5.66	33	2	6.06
Oyarifa	44	11	25.00	30	5	16.67
T. Hill	12	5	41.67	8	2	25.00
Teshie	29	8	27.59	19	5	26.32
Tuba	88	27	30.68	68	8	11.76
Total	351	77	21.94	238	32	13.45

**Table 6:** Entomological transmission indices of host-seeking *An. gambiae* s.l. across various study sites.

Site Category	Study sites	HBR	Samples tested	Sporozoite positive	SIR	EIR/year
**LS**	**Nima**	6.9	62	1	0.016	40.621
	**Chorkor**	5.6	59	2	0.034	69.288
**MS**	**Teshie**	37.3	200	3	0.015	204.218
	**Dansoman**	22.6	121	7	0.058	477.215
**HS**	**East Legon**	51.4	377	10	0.027	497.639
	**Tantra Hill**	44.7	216	1	0.005	75.535
**IUF**	**Opeibea**	36.6	230	5	0.022	290.413
	**Tuba**	176.5	649	10	0.015	992.643
**PU**	**Oyarifa**	47.1	378	10	0.026	454.802
	**Medie**	30.8	184	8	0.043	488.783

LS = Low socioeconomic, MS = Middle socioeconomic, HS = High socioeconomic, IUF = Irrigated urban farming, PU = Peri-urban, HBR= Human biting rate, SIR = Sporozoite infection rate, EIR = Entomological inoculation rate.

**Table 7:** Frequency distribution of kdr L1014F, L1014S and Ace-1 G119S among resting *An. gambiae* s.l.

	N	L1014F	n	F(L1014S)	N	L1014S	n	F(L1014S)	N	G119S	n	F (G119S)
Indoor	351	RR	331	1.0	351	RR	0	0	351	RR	148	0.7
		RS	20			RS	59			RS	177	
		SS	0			SS	292			SS	26	
Outdoor	238	RR	222	1.0	238	RR	0	0	238	RR	82	0.6
		RS	16			RS	33			RS	135	
		SS	0			SS	205			SS	21	
Species												
An. coluzzii	380	RR	362	1.0	380	RR	0	0	380	RR	146	0.7
		RS	18			RS	60			RS	219	
		SS	0			SS	320			SS	15	
An. gambiae s.s.	159	RR	142	0.9	159	RR	0	0.1	159	RR	74	0.7
		RS	17			RS	19			RS	64	
		SS	0			SS	140			SS	21	
Hybrid	50	RR	49	1.0	50	RR	0	0.1	50	RR	10	0.5
		RS	1			RS	13			RS	29	
		SS	0			SS	37			SS	11	

F(kdr) = 2RR + RS/2n Ahadji-Dabla 2019. F: allelic frequency, N = number of samples tested, n = total number of samples positive for a specific genotype.

## Data Availability

The datasets utilized and analyzed in this study is available and can be obtained from the corresponding author upon request.

## Abbreviations

LS: Lower-socioeconomic status
MS: Middle-socioeconomic status
HS: High-socioeconomic status
PU: Peri-urban
IUF: Irrigated urban farming
PCR: Polymerase chain reaction
LSM: Larval source management
RFLP: Restriction fragment length polymorphism
HBR: Human biting rate
EIR: Entomological inoculation rate
SIR: Sporozoite infection rate
HLC: Human landing catches
